# (*Z*)-*N*-(2,6-Diiso­propyl­phen­yl)-4-nitro­benzimidoyl chloride

**DOI:** 10.1107/S1600536813020862

**Published:** 2013-08-07

**Authors:** Gamal A. El-Hiti, Keith Smith, Dyfyr Heulyn Jones, Ali Masmali, Benson M. Kariuki

**Affiliations:** a Department of Optometry, College of Applied Medical Sciences, King Saud University, PO Box 10219, Riyadh 11433, Saudi Arabia; b School of Chemistry, Cardiff University, Main Building, Park Place, Cardiff CF10 3AT, Wales

## Abstract

In the title compound, C_19_H_21_ClN_2_O_2_, the aromatic rings are approximately perpendicular to each other, subtending a dihedral angle of 87.7 (1)°. In the crystal, the 4-nitro­phenyl groups of pairs of neighbouring mol­ecules are parallel and oriented head-to-tail with a ring centroid–centroid distance of 3.9247 (12) Å, leading to a π–π inter­action between the pair. The faces of each phenyl ring of the 2,6-diiso­propyl­phenyl group inter­act with two different groups, *viz.* a chloro group of an adjacent mol­ecule on one side and the edge of the 4-nitro­phenyl ring of a second mol­ecule on the other side.

## Related literature

For the synthesis and applications of imidoyl chlorides, see: Pelter *et al.* (1975 ▶); Manley & Bilodeau (2002 ▶); Cunico & Pandey (2005 ▶); Raussukana *et al.* (2006 ▶); Zheng & Alper (2008 ▶); Kuszpit *et al.* (2011 ▶). For a related structure of an imidoyl chloride, see: Seidelmann *et al.* (1998 ▶).
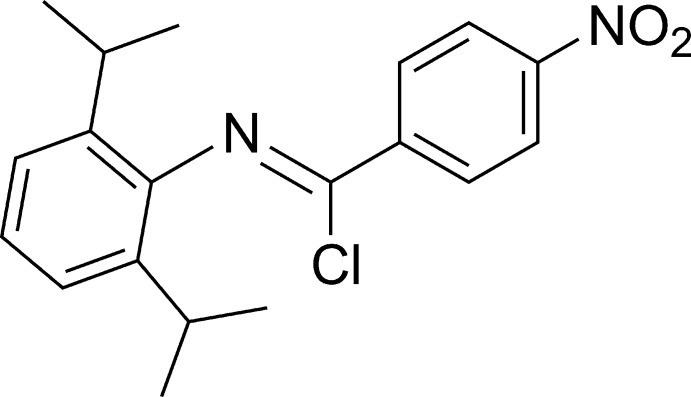


## Experimental

### 

#### Crystal data

C_19_H_21_ClN_2_O_2_
*M* *_r_* = 344.83
Triclinic, 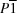

*a* = 8.2988 (4) Å
*b* = 10.4667 (3) Å
*c* = 10.9665 (3) Å
α = 75.568 (2)°
β = 85.411 (2)°
γ = 74.145 (2)°
*V* = 887.33 (6) Å^3^
*Z* = 2
Mo *K*α radiation
μ = 0.23 mm^−1^
*T* = 150 K
0.35 × 0.20 × 0.15 mm

#### Data collection

Nonius KappaCCD diffractometer
Absorption correction: multi-scan (*DENZO*/*SCALEPACK*; Otwinowski & Minor, 1997 ▶) *T*_min_ = 0.924, *T*_max_ = 0.967
6021 measured reflections
4232 independent reflections
3108 reflections with *I* > 2σ(*I*)
*R*_int_ = 0.034

#### Refinement

*R*[*F* ^2^ > 2σ(*F* ^2^)] = 0.062
*wR*(*F* ^2^) = 0.173
*S* = 1.06
4232 reflections
222 parameters
H-atom parameters constrained
Δρ_max_ = 0.32 e Å^−3^
Δρ_min_ = −0.41 e Å^−3^

### 

Data collection: *COLLECT* (Nonius, 2000 ▶); cell refinement: *DENZO*/*SCALEPACK* (Otwinowski & Minor, 1997 ▶); data reduction: *DENZO*/*SCALEPACK*; program(s) used to solve structure: *SIR92* (Altomare *et al.*, 1993 ▶); program(s) used to refine structure: *SHELXL97* (Sheldrick, 2008 ▶); molecular graphics: *ORTEP99* for Windows (Farrugia, 2012 ▶) and *Mercury* (Macrae *et al.*, 2006 ▶); software used to prepare material for publication: *WinGX* (Farrugia, 2012 ▶).

## Supplementary Material

Crystal structure: contains datablock(s) I, New_Global_Publ_Block. DOI: 10.1107/S1600536813020862/is5293sup1.cif

Structure factors: contains datablock(s) I. DOI: 10.1107/S1600536813020862/is5293Isup2.hkl

Supplementary material file. DOI: 10.1107/S1600536813020862/is5293Isup3.cml

Additional supplementary materials:  crystallographic information; 3D view; checkCIF report

## Figures and Tables

**Table 1:** Hydrogen-bond geometry (Å, °) *Cg*1 and *Cg*2 are the centroids of the C1–C6 and C8–C13 rings, respectively.

*D*—H⋯*A*	*D*—H	H⋯*A*	*D*⋯*A*	*D*—H⋯*A*
C6—H6⋯*Cg*2^i^	0.95	2.67	3.511 (2)	147
C16—H16*B*⋯*Cg*1^ii^	0.98	2.79	3.663 (3)	149

Symmetry codes: (i) 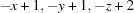; (ii) 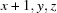.

**Fig. 1.**
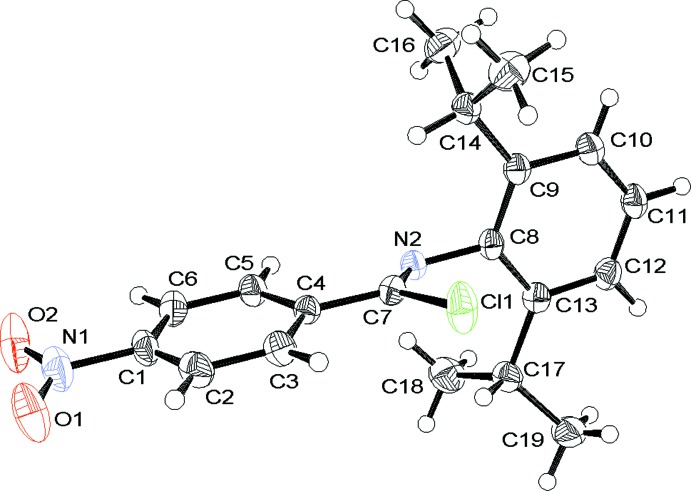
Molecular structure of the title compound, showing atom labels and 50% probability displacement ellipsoids for non-H atoms.

**Fig. 2.**
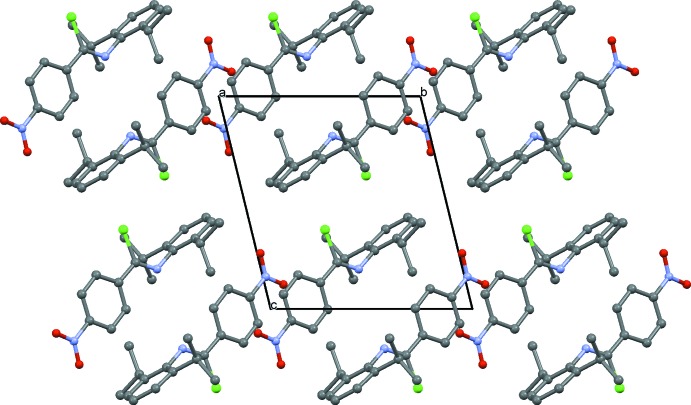
A packing view of the title compound along the a axis.

**Fig. 3.**
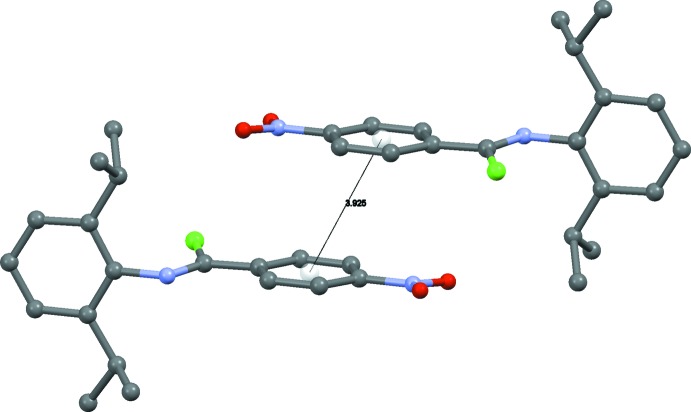
A pair of molecules showing the ring centroid-centroid distance for parallel 4-nitrobenzyl groups.

**Fig. 4.**
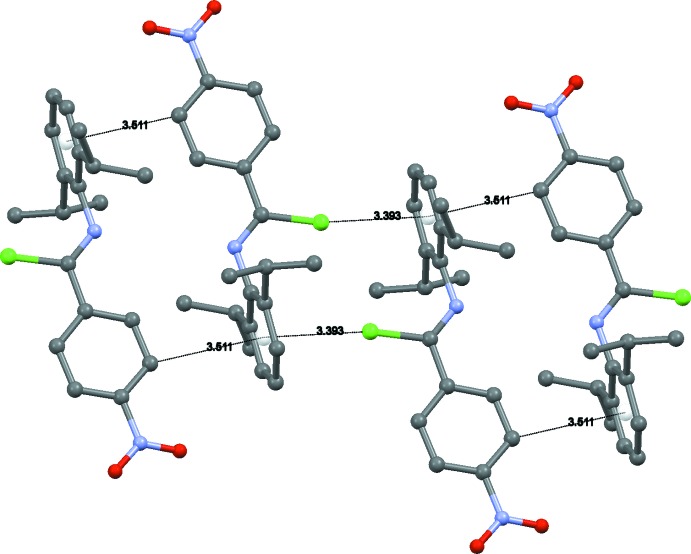
A segment showing edge-to-face and chloro-to-face contacts in the crystal structure.

## supplementary crystallographic information

### 1. Comment

The title compound I, a useful synthetic intermediate, was synthesized in good yield by the reaction of *N*-(2,6-diisopropylphenyl)-4-nitrobenzamide with phosphorus pentachloride. Imidoyl chlorides are useful reactive intermediates in syntheses of ketones from trialkylcyanoborates (Pelter *et al.*, 1975), of highly substituted 2-imidazolines *via* a ring-expansion reaction with aziridines (Kuszpit *et al.*, 2011), and by *in situ* reaction with pyridine-1-oxides to give 2-aminopyridine amides (Manley *et al.*, 2002). They have also been used as precursors to α-iminoamides (Cunico *et al.*, 2005), isoquinolin-1(2*H*)-ones *via* a palladium-catalyzed reaction with diethyl(2-iodoaryl)malonates (Zheng *et al.*, 2008), and 1,3-oxathiolanones and benzoxathianones by reaction with mercaptocarboxylic acids (Raussukana *et al.*, 2006). The X-ray crystal structure of *N*-(diethylaminothiocarbonyl)ferrocenecarbimidoyl chloride has been reported (Seidelmann *et al.*, 1998).

In the molecule (Fig. 1), the aromatic rings of the 2,6-diisopropylphenyl and 4-nitrophenyl groups are approximately perpendicular to each other; the dihedral angle between the least-squares planes through the rings is 87.7 (1)°. The molecule has no strong hydrogen bond donor and the crystal structure is shown in Figure 2. The 4-nitrophenyl groups of neighboring molecules are parallel and oriented head-to-tail with a ring centroid-centroid distance of 3.9247 (12) Å, leading to a π–π interaction (Fig. 3). One face of the phenyl ring of the 2,6-diisopropylphenyl group interacts with the chloro group of an adjacent molecule (C7—Cl1···*Cg*2) and the other face of the same ring interacts with the edge of the 4-nitrophenyl ring of a second molecule (C6—H6···*Cg*2; Fig. 4); *Cg*2 is the centroid of the C8–C13 ring. Another interaction, C16—H16B···*Cg*1, is also observed; *Cg*1 is the centroid of the C1–C6 ring.

### 2. Experimental

Synthesis of*N*-(2,6-diisopropylphenyl)-4-nitrobenzimidoyl chloride (I)

An oven dried two necked 100 ml flask equipped with a magnetic stirrer, septum-capped reflux condenser and septum was flushed with N_2_ and phosphorus pentachloride (4.42 g, 21 mmol) and dry toluene (40 ml) were added. The mixture was stirred for 5 min then *N*-(2,6-diisopropylphenyl)-4-nitrobenzamide (6.90 g, 21 mmol) was quickly added to the flask under a fast stream of N_2_, and the septum replaced by a stopper. The mixture was heated to reflux for 2 h, whereupon it became homogeneous and gas evolution was observed. Phosphorus oxychloride and toluene were removed under reduced pressure and the crude product was quickly extracted with hot diethyl ether (3 × 80 ml). The diethyl ether washings were evaporated under a fast stream of N_2_ overnight, during which process bright yellow prisms of *N*-(2,6-diisopropylphenyl)-4-nitrobenzimidoyl chloride (6.93 g, 95%) separated; m.p. 144–146 °C. HREI^+^–MS m/*z*: calcd for C_19_H_21_N_2_O_2_^35^Cl 344.1292, found 344.1301.

### 3. Refinement

H atoms were positioned geometrically (C—H = 0.95–1.00 Å) and refined using a riding model with *U*_iso_(H) = 1.2*U*_eq_(C) or 1.5*U*_eq_(methyl C), allowing for free rotation of the methyl groups about the C—C bond.

### Crystal data

**Table:**

C_19_H_21_ClN_2_O_2_	*Z* = 2
*M**_r_* = 344.83	*F*(000) = 364
Triclinic, *P*1	*D*_x_ = 1.291 Mg m^−^^3^
Hall symbol: -P 1	Mo *K*α radiation, λ = 0.71073 Å
*a* = 8.2988 (4) Å	Cell parameters from 3108 reflections
*b* = 10.4667 (3) Å	θ = 2.8–28.3°
*c* = 10.9665 (3) Å	µ = 0.23 mm^−^^1^
α = 75.568 (2)°	*T* = 150 K
β = 85.411 (2)°	Block, yellow
γ = 74.145 (2)°	0.35 × 0.20 × 0.15 mm
*V* = 887.33 (6) Å^3^	

### Data collection

**Table:**

Nonius KappaCCD diffractometer	4232 independent reflections
Radiation source: fine-focus sealed tube	3108 reflections with *I* > 2σ(*I*)
Graphite monochromator	*R*_int_ = 0.034
ω and φ scans	θ_max_ = 28.3°, θ_min_ = 2.8°
Absorption correction: multi-scan (*DENZO*/*SCALEPACK*; Otwinowski & Minor, 1997)	*h* = −11→10
*T*_min_ = 0.924, *T*_max_ = 0.967	*k* = −13→13
6021 measured reflections	*l* = −14→14

### Refinement

**Table:**

Refinement on *F*^2^	Secondary atom site location: difference Fourier map
Least-squares matrix: full	Hydrogen site location: inferred from neighbouring sites
*R*[*F*^2^ > 2σ(*F*^2^)] = 0.062	H-atom parameters constrained
*wR*(*F*^2^) = 0.173	*w* = 1/[σ^2^(*F*_o_^2^) + (0.0765*P*)^2^ + 0.589*P*] where *P* = (*F*_o_^2^ + 2*F*_c_^2^)/3
*S* = 1.06	(Δ/σ)_max_ = 0.005
4232 reflections	Δρ_max_ = 0.32 e Å^−^^3^
222 parameters	Δρ_min_ = −0.41 e Å^−^^3^
0 restraints	Extinction correction: *SHELXL97* (Sheldrick, 2008), Fc^*^=kFc[1+0.001xFc^2^λ^3^/sin(2θ)]^-1/4^
Primary atom site location: structure-invariant direct methods	Extinction coefficient: 0.094 (10)

### Special details

**Table:**

Experimental. ^1^H (400 MHz; CDCl_3_) δ: 8.25 (2 H, d, *J* = 8.4 Hz), 8.17 (2 H, d, *J* = 8.4 Hz), 7.05–7.12 (2 H, m), 6.97–7.04 (1 H, m), 2.66 (2 H, app. sept, *J* = 6.9 Hz), 1.11 (6 H, d, *J* = 6.6 Hz), 1.05 (6 H, d, *J* = 6.6 Hz) – the two 6 H doublets coalesced at 50 °C; ^13^C (125 MHz; CDCl_3_) δ: 149.9 (*s*), 143.4 (*s*), 141.8 (*s*), 140.1 (*s*), 136.3 (*s*), 130.3 (*d*), 125.5 (*d*), 123.7 (*d*), 123.3 (*d*), 28.8 (*d*), 23.3 (*q*), 22.8 (*q*); v_max_ (thin film/cm^-1^): 3017, 2966, 2929, 2871, 1662, 1605, 1529, 1349, 1216, 1168, 1461.
Geometry. All s.u.'s (except the s.u. in the dihedral angle between two l.s. planes) are estimated using the full covariance matrix. The cell s.u.'s are taken into account individually in the estimation of s.u.'s in distances, angles and torsion angles; correlations between s.u.'s in cell parameters are only used when they are defined by crystal symmetry. An approximate (isotropic) treatment of cell s.u.'s is used for estimating s.u.'s involving l.s. planes.
Refinement. Refinement of *F*^2^ against ALL reflections. The weighted *R*-factor *wR* and goodness of fit *S* are based on *F*^2^, conventional *R*-factors *R* are based on *F*, with *F* set to zero for negative *F*^2^. The threshold expression of *F*^2^ > 2σ(*F*^2^) is used only for calculating *R*-factors(gt) *etc*. and is not relevant to the choice of reflections for refinement. *R*-factors based on *F*^2^ are statistically about twice as large as those based on *F*, and *R*- factors based on ALL data will be even larger.

### Fractional atomic coordinates and isotropic or equivalent isotropic displacement parameters (Å^2^)

**Table:**

	*x*	*y*	*z*	*U*_iso_*/*U*_eq_	
C1	0.2830 (3)	0.0882 (2)	1.0555 (2)	0.0319 (5)	
C2	0.2874 (3)	0.0814 (2)	0.9309 (2)	0.0343 (5)	
H2	0.2496	0.0132	0.9075	0.041*	
C3	0.3483 (3)	0.1763 (2)	0.8410 (2)	0.0299 (5)	
H3	0.3551	0.1720	0.7552	0.036*	
C4	0.3996 (2)	0.27816 (19)	0.87595 (19)	0.0234 (4)	
C5	0.3944 (3)	0.2816 (2)	1.0028 (2)	0.0277 (5)	
H5	0.4310	0.3500	1.0269	0.033*	
C6	0.3360 (3)	0.1857 (2)	1.0938 (2)	0.0320 (5)	
H6	0.3326	0.1873	1.1803	0.038*	
C7	0.4587 (3)	0.3856 (2)	0.78235 (18)	0.0237 (4)	
C8	0.5521 (3)	0.5859 (2)	0.72503 (18)	0.0239 (4)	
C9	0.7254 (3)	0.5618 (2)	0.70425 (19)	0.0259 (4)	
C10	0.7851 (3)	0.6667 (2)	0.6259 (2)	0.0306 (5)	
H10	0.9022	0.6527	0.6104	0.037*	
C11	0.6771 (3)	0.7907 (2)	0.5702 (2)	0.0317 (5)	
H11	0.7201	0.8614	0.5180	0.038*	
C12	0.5055 (3)	0.8114 (2)	0.5911 (2)	0.0306 (5)	
H12	0.4321	0.8961	0.5513	0.037*	
C13	0.4386 (3)	0.7111 (2)	0.6689 (2)	0.0267 (5)	
C14	0.8468 (3)	0.4269 (2)	0.7645 (2)	0.0307 (5)	
H14	0.7792	0.3633	0.8100	0.037*	
C15	0.9545 (3)	0.3613 (3)	0.6651 (3)	0.0427 (6)	
H15A	0.8819	0.3544	0.6022	0.064*	
H15B	1.0214	0.2697	0.7057	0.064*	
H15C	1.0293	0.4178	0.6239	0.064*	
C16	0.9550 (3)	0.4460 (3)	0.8613 (2)	0.0406 (6)	
H16A	1.0282	0.5032	0.8185	0.061*	
H16B	1.0235	0.3566	0.9054	0.061*	
H16C	0.8827	0.4905	0.9222	0.061*	
C17	0.2510 (3)	0.7304 (2)	0.6917 (2)	0.0308 (5)	
H17	0.2250	0.6450	0.6838	0.037*	
C18	0.2005 (3)	0.7468 (3)	0.8252 (3)	0.0446 (6)	
H18A	0.2680	0.6693	0.8861	0.067*	
H18B	0.0816	0.7497	0.8397	0.067*	
H18C	0.2195	0.8320	0.8353	0.067*	
C19	0.1440 (3)	0.8492 (3)	0.5950 (3)	0.0438 (6)	
H19A	0.1614	0.9356	0.6032	0.066*	
H19B	0.0255	0.8510	0.6101	0.066*	
H19C	0.1766	0.8370	0.5099	0.066*	
N1	0.2200 (3)	−0.0141 (2)	1.1512 (2)	0.0457 (6)	
N2	0.4892 (2)	0.48451 (17)	0.81255 (16)	0.0246 (4)	
O1	0.1607 (3)	−0.0925 (2)	1.1148 (2)	0.0716 (7)	
O2	0.2325 (3)	−0.0162 (2)	1.2616 (2)	0.0651 (6)	
Cl1	0.48157 (10)	0.36574 (7)	0.62743 (5)	0.0461 (2)	

### Atomic displacement parameters (Å^2^)

**Table:**

	*U*^11^	*U*^22^	*U*^33^	*U*^12^	*U*^13^	*U*^23^
C1	0.0304 (11)	0.0202 (10)	0.0391 (12)	−0.0059 (8)	0.0081 (9)	0.0008 (9)
C2	0.0350 (12)	0.0224 (10)	0.0473 (14)	−0.0115 (9)	0.0001 (10)	−0.0074 (9)
C3	0.0334 (11)	0.0270 (11)	0.0312 (11)	−0.0100 (9)	0.0017 (9)	−0.0085 (9)
C4	0.0235 (10)	0.0192 (9)	0.0265 (10)	−0.0061 (7)	0.0001 (8)	−0.0031 (8)
C5	0.0318 (11)	0.0257 (10)	0.0269 (10)	−0.0105 (8)	0.0008 (9)	−0.0056 (8)
C6	0.0360 (12)	0.0272 (11)	0.0284 (11)	−0.0063 (9)	0.0046 (9)	−0.0023 (9)
C7	0.0267 (10)	0.0229 (9)	0.0205 (9)	−0.0056 (8)	−0.0003 (8)	−0.0043 (7)
C8	0.0308 (11)	0.0219 (9)	0.0210 (9)	−0.0102 (8)	0.0014 (8)	−0.0056 (7)
C9	0.0302 (11)	0.0233 (10)	0.0249 (10)	−0.0092 (8)	0.0009 (8)	−0.0050 (8)
C10	0.0328 (11)	0.0293 (11)	0.0304 (11)	−0.0125 (9)	0.0025 (9)	−0.0045 (9)
C11	0.0396 (12)	0.0267 (11)	0.0296 (11)	−0.0154 (9)	0.0027 (9)	−0.0017 (8)
C12	0.0359 (12)	0.0241 (10)	0.0296 (11)	−0.0086 (9)	−0.0027 (9)	−0.0010 (8)
C13	0.0312 (11)	0.0247 (10)	0.0260 (10)	−0.0095 (8)	0.0000 (8)	−0.0070 (8)
C14	0.0301 (11)	0.0264 (11)	0.0326 (11)	−0.0076 (9)	0.0018 (9)	−0.0018 (9)
C15	0.0432 (14)	0.0384 (13)	0.0435 (14)	−0.0016 (11)	−0.0010 (11)	−0.0143 (11)
C16	0.0391 (13)	0.0400 (13)	0.0367 (13)	−0.0010 (10)	−0.0049 (11)	−0.0070 (10)
C17	0.0302 (11)	0.0239 (10)	0.0377 (12)	−0.0081 (8)	−0.0002 (9)	−0.0051 (9)
C18	0.0389 (14)	0.0480 (15)	0.0462 (15)	−0.0086 (11)	0.0087 (11)	−0.0156 (12)
C19	0.0318 (12)	0.0367 (13)	0.0554 (16)	−0.0076 (10)	−0.0063 (11)	0.0028 (11)
N1	0.0472 (13)	0.0264 (10)	0.0566 (15)	−0.0112 (9)	0.0153 (11)	−0.0006 (10)
N2	0.0272 (9)	0.0228 (8)	0.0239 (8)	−0.0092 (7)	0.0009 (7)	−0.0035 (7)
O1	0.0921 (18)	0.0478 (12)	0.0828 (17)	−0.0473 (12)	0.0134 (14)	−0.0024 (11)
O2	0.0942 (17)	0.0485 (12)	0.0469 (12)	−0.0290 (12)	0.0254 (12)	0.0019 (9)
Cl1	0.0793 (5)	0.0479 (4)	0.0234 (3)	−0.0365 (3)	0.0067 (3)	−0.0110 (2)

### Geometric parameters (Å, º)

**Table:**

C1—C6	1.379 (3)	C12—C13	1.391 (3)
C1—C2	1.382 (3)	C12—H12	0.9500
C1—N1	1.477 (3)	C13—C17	1.522 (3)
C2—C3	1.386 (3)	C14—C16	1.526 (3)
C2—H2	0.9500	C14—C15	1.529 (3)
C3—C4	1.393 (3)	C14—H14	1.0000
C3—H3	0.9500	C15—H15A	0.9800
C4—C5	1.397 (3)	C15—H15B	0.9800
C4—C7	1.485 (3)	C15—H15C	0.9800
C5—C6	1.388 (3)	C16—H16A	0.9800
C5—H5	0.9500	C16—H16B	0.9800
C6—H6	0.9500	C16—H16C	0.9800
C7—N2	1.254 (3)	C17—C18	1.529 (4)
C7—Cl1	1.752 (2)	C17—C19	1.533 (3)
C8—C9	1.400 (3)	C17—H17	1.0000
C8—C13	1.414 (3)	C18—H18A	0.9800
C8—N2	1.427 (3)	C18—H18B	0.9800
C9—C10	1.396 (3)	C18—H18C	0.9800
C9—C14	1.520 (3)	C19—H19A	0.9800
C10—C11	1.385 (3)	C19—H19B	0.9800
C10—H10	0.9500	C19—H19C	0.9800
C11—C12	1.390 (3)	N1—O2	1.218 (3)
C11—H11	0.9500	N1—O1	1.221 (3)
			
C6—C1—C2	122.8 (2)	C9—C14—C15	111.36 (19)
C6—C1—N1	118.8 (2)	C16—C14—C15	111.3 (2)
C2—C1—N1	118.4 (2)	C9—C14—H14	107.7
C1—C2—C3	118.5 (2)	C16—C14—H14	107.7
C1—C2—H2	120.7	C15—C14—H14	107.7
C3—C2—H2	120.7	C14—C15—H15A	109.5
C2—C3—C4	120.3 (2)	C14—C15—H15B	109.5
C2—C3—H3	119.9	H15A—C15—H15B	109.5
C4—C3—H3	119.9	C14—C15—H15C	109.5
C3—C4—C5	119.64 (19)	H15A—C15—H15C	109.5
C3—C4—C7	122.22 (19)	H15B—C15—H15C	109.5
C5—C4—C7	118.14 (18)	C14—C16—H16A	109.5
C6—C5—C4	120.5 (2)	C14—C16—H16B	109.5
C6—C5—H5	119.7	H16A—C16—H16B	109.5
C4—C5—H5	119.7	C14—C16—H16C	109.5
C1—C6—C5	118.2 (2)	H16A—C16—H16C	109.5
C1—C6—H6	120.9	H16B—C16—H16C	109.5
C5—C6—H6	120.9	C13—C17—C18	111.42 (19)
N2—C7—C4	121.94 (18)	C13—C17—C19	113.50 (19)
N2—C7—Cl1	122.36 (16)	C18—C17—C19	110.2 (2)
C4—C7—Cl1	115.70 (15)	C13—C17—H17	107.1
C9—C8—C13	122.16 (19)	C18—C17—H17	107.1
C9—C8—N2	118.85 (17)	C19—C17—H17	107.1
C13—C8—N2	118.84 (18)	C17—C18—H18A	109.5
C10—C9—C8	117.86 (19)	C17—C18—H18B	109.5
C10—C9—C14	120.20 (19)	H18A—C18—H18B	109.5
C8—C9—C14	121.94 (18)	C17—C18—H18C	109.5
C11—C10—C9	121.3 (2)	H18A—C18—H18C	109.5
C11—C10—H10	119.3	H18B—C18—H18C	109.5
C9—C10—H10	119.3	C17—C19—H19A	109.5
C10—C11—C12	119.7 (2)	C17—C19—H19B	109.5
C10—C11—H11	120.2	H19A—C19—H19B	109.5
C12—C11—H11	120.2	C17—C19—H19C	109.5
C11—C12—C13	121.6 (2)	H19A—C19—H19C	109.5
C11—C12—H12	119.2	H19B—C19—H19C	109.5
C13—C12—H12	119.2	O2—N1—O1	124.1 (2)
C12—C13—C8	117.3 (2)	O2—N1—C1	118.0 (2)
C12—C13—C17	122.60 (19)	O1—N1—C1	117.9 (2)
C8—C13—C17	120.03 (18)	C7—N2—C8	122.75 (18)
C9—C14—C16	110.82 (19)		

### Hydrogen-bond geometry (Å, º)

*Cg*1 and *Cg*2 are the centroids of the C1–C6 and C8–C13 rings, respectively.

**Table:**

*D*—H···*A*	*D*—H	H···*A*	*D*···*A*	*D*—H···*A*
C6—H6···*Cg*2^i^	0.95	2.67	3.511 (2)	147
C16—H16*B*···*Cg*1^ii^	0.98	2.79	3.663 (3)	149

Symmetry codes: (i) −*x*+1, −*y*+1, −*z*+2; (ii) *x*+1, *y*, *z*.
